# The effects of thermal acclimation on cardio-respiratory performance in an Antarctic fish (*Notothenia coriiceps*)

**DOI:** 10.1093/conphys/coy069

**Published:** 2018-12-13

**Authors:** William Joyce, Michael Axelsson, Stuart Egginton, Anthony P Farrell, Elizabeth L Crockett, Kristin M O’Brien

**Affiliations:** 1 Department of Zoophysiology, Aarhus University, Aarhus C, Denmark; 2 Department of Biological and Environmental Sciences, University of Gothenburg, Gothenburg, Sweden; 3 School of Biomedical Sciences, University of Leeds, Leeds, UK; 4 Department of Zoology, University of British Columbia, Vancouver, BC, Canada; 5 Department of Biological Sciences, Ohio University, Athens, OH, USA; 6 Institute of Arctic Biology, University of Alaska Fairbanks, Fairbanks, AK, USA

**Keywords:** Blood flow, heart rate, notothenioid fish, oxygen consumption, temperature

## Abstract

The Southern Ocean has experienced stable, cold temperatures for over 10 million years, yet particular regions are currently undergoing rapid warming. To investigate the impacts of warming on cardiovascular oxygen transport, we compared the cardio-respiratory performance in an Antarctic notothenioid (*Notothenia coriiceps*) that was maintained at 0 or 5°C for 6.0–9.5 weeks. When compared at the fish’s respective acclimation temperature, the oxygen consumption rate and cardiac output were significantly higher in 5°C-acclimated than 0°C-acclimated fish. The 2.7-fold elevation in cardiac output in 5°C-acclimated fish (17.4 *vs*. 6.5 ml min^−1^ kg^−1^) was predominantly due to a doubling of stroke volume, likely in response to increased cardiac preload, as measured by higher central venous pressure (0.15 *vs*. 0.08 kPa); tachycardia was minor (29.5 *vs*. 25.2 beats min^−1^). When fish were acutely warmed, oxygen consumption rate increased by similar amounts in 0°C- and 5°C-acclimated fish at equivalent test temperatures. In both acclimation groups, the increases in oxygen consumption rate during acute heating were supported by increased cardiac output achieved by elevating heart rate, while stroke volume changed relatively little. Cardiac output was similar between both acclimation groups until 12°C when cardiac output became significantly higher in 5°C-acclimated fish, driven largely by their higher stroke volume. Although cardiac arrhythmias developed at a similar temperature (~14.5°C) in both acclimation groups, the hearts of 5°C-acclimated fish continued to pump until significantly higher temperatures (CT_max_ for cardiac function 17.7 *vs.* 15.0°C for 0°C-acclimated fish). These results demonstrate that *N. coriiceps* is capable of increasing routine cardiac output during both acute and chronic warming, although the mechanisms are different (heart rate-dependent *versus* primarily stroke volume-dependent regulation, respectively). Cardiac performance was enhanced at higher temperatures following 5°C acclimation, suggesting cardiovascular function may not constrain the capacity of *N. coriiceps* to withstand a warming climate.

## Teaser

In *Notothenia coriiceps*, acclimation to increased temperature (5 *vs.* 0°C for 6.0–9.5 weeks) increased upper thermal limits of the cardiovascular system during acute heating. Whilst cardiac output increased with acute heating as a result of elevated heart rate, after chronic warming cardiac output was surprisingly increased primarily by an elevation in stroke volume.

## Introduction

The Southern Ocean has been characterised by stable, frigid temperatures (−1.9 to +1.5°C; Littlepage, 1965; Zachos *et al.*, 2001) for over 10 million years that may have resulted in the evolution of stenothermy in its ectothermic inhabitants (Peck *et al.*, 2014; Beers and Jayasundara, 2015). However, some areas, particularly the Western Antarctic Peninsula region, are currently experiencing some of the most dramatic effects of climate warming in which average surface water temperatures have risen by ~1°C over the past 50 years (Meredith and King, 2005; Steig *et al.*, 2009). This rate is expected to continue over the next century (IPCC, 2014; Ashton *et al.* 2017). Physiological studies to assess how Antarctic marine organisms, including fishes, respond to increased temperature are therefore paramount to inform predictive models and evaluate the future fate of ecosystems in the Southern Ocean.

Tolerance to increased temperature is notoriously low in Antarctic fishes, which show the lowest upper thermal limits of any fish (e.g. Somero and DeVries, 1967). However, recent studies have suggested upper thermal tolerance can be improved following a period (days to weeks) of acclimation to a 4°C elevation from ambient temperatures (Podrabsky and Somero, 2006; Bilyk and DeVries, 2011). The previous studies measuring absolute thermal limits (e.g. Podrabsky and Somero, 2006; Bilyk and DeVries, 2011) have typically used behavioural criteria, such as loss of righting reflex, to determine upper critical thermal maxima (CT_MAX_). It is also clear that the robustness of cardiovascular function is a key component of thermal tolerance in fishes (e.g. Farrell *et al.*, 2009; Casselman *et al.*, 2012; Ferreira *et al.*, 2014; Farrell, 2016). Indeed, in *Pagothenia borchgrevinki*, an Antarctic teleost, 4°C acclimation resulted in the factorial scope for cardiac output being highest at elevated temperature (8°C; Seebacher *et al.*, 2005; Franklin *et al.*, 2007). Franklin *et al.* (2007), however, observed that maximum cardiac output was unchanged by warm acclimation, and that in both −1°C- and 4°C-acclimated fish, maximum cardiac output was unchanged from −1°C to 8°C. In *Trematomus bernacchii*, 4.5°C acclimation did not alter the temperature at which cardiac arrhythmia develops (*T*_arr_) during acute warming (Jayasundara *et al.*, 2013), which is in contrast to similar studies in temperate fishes such as *Gillichthys mirabilis* (Jayasundara and Somero, 2013), *Carassius auratus* (Ferreira *et al.*, 2014) and *Rutilus rutilus* (Badr *et al.*, 2016), in which warm acclimation increased *T*_arr_.

Robinson and Davison (2008) reported that *P. borchgrevinki* acclimated at 4°C for 4 weeks had similar rates of oxygen consumption (*Ṁ*_O2_) to those maintained at −1°C measured at the respective temperatures. However, Egginton and Campbell (2016) observed no acclimatory compensation (i.e. downregulation) of heart rate (*ƒ*_H_) or *Ṁ*_O2_ in *N. coriiceps* acclimated at 5°C for 6 weeks when compared to individuals acutely warmed to the same temperature. Likewise, Strobel *et al.* (2012) observed only partial down-regulation of *Ṁ*_O2_ in 7°C-acclimated *Notothenia rossii* after 4–5 weeks. Recently, Enzor *et al.* (2017) confirmed that *P. borchgrevinki* is capable of compensating *Ṁ*_O2_ within 6 weeks of acclimation to 4°C but observed that in *T. bernacchii*, a more benthic species, *Ṁ*_O2_ was not compensated after 8 weeks of acclimation to 4°C. Yet, Sandersfeld *et al.* (2015) observed a clearly down-regulated *Ṁ*_O2_ in *T. bernacchii* after a 9-week acclimation period. Enzor *et al.* (2017), thus, concluded that this species requires 8–9 weeks to acclimate to increased temperature.

In the present study, we expanded upon the work of Egginton and Campbell (2016), which only measured *ƒ*_H_ and *Ṁ*_O2_ after 6 weeks of acclimation, by comparing *N. coriiceps* exposed to either 0 or 5°C for a longer period of time (6.0–9.5 weeks). Furthermore, we measured an extended suite of cardiovascular parameters, including cardiac output (*Q̇*), central venous pressure (*P*_cv_), and haematocrit (Hct), along with *Ṁ*_O2_ and *ƒ*_H_, at these two acclimation temperatures. We hypothesised that, following acclimation, the chronic increase in *Ṁ*_O2_ at 5°C, in comparison to fish acclimated to 0°C, would be associated with increased *Q̇* as a result of elevated *ƒ*_H_ as opposed to changes in cardiac stroke volume (*V*_S_) (Egginton and Campbell, 2016). We next investigated cardio-respiratory performance during an acute thermal challenge in which temperature was increased from 0°C to well above 10°C when cardiac failure occurred. We hypothesised that increased CT_MAX_ in 5°C-acclimated fish (Bilyk and DeVries, 2011) would be associated with increasing *Q̇* to a higher critical temperature and that a higher *Q̇* would be attained at elevated temperature (below CT_MAX_).

## Materials and methods

### Experimental animals

*Notothenia coriiceps* of both sexes were caught in baited pot traps and otter trawls deployed in Dallmann Bay (64°08′S, 62°40′W) and in the vicinity of Low Island (63°30′S, 62°42′W) from the *ARSV Laurence M. Gould* and maintained onboard for up to 3 days in flow-through seawater tanks. The fish were transported to the US Antarctic research base Palmer Station (Anvers Island) where they were maintained in covered, circulating seawater tanks. After 3 days of recovery from the stress of capture and transportation, the fish were randomly assigned to 700 or 1700-l tanks designated to be at 5 ± 1°C (5°C acclimated) or 0 ± 1°C (0°C acclimated). The tanks used for 5°C acclimation were heated from 0°C at a rate of 1°C day^−1^ and then held at 5°C for a minimum of 6.0 and up to 9.5 weeks. This acclimation period is longer than previous work on this species (Egginton and Campbell, 2016) and allowed us to gain insight into the dynamic nature of thermal acclimation in this species. The 0°C-acclimated fish were maintained in captivity for the same duration and the experiments were conducted in parallel. The present study used seven 0°C-acclimated and six 5°C-acclimated fish. Fish were fed *ad libitum* chopped fish muscle every 2 days. Antarctic fish are known to exhibit a small peak specific dynamic action (the metabolic response to feeding) that is very long lasting (1–2 weeks) (Sandblom *et al.*, 2012), so no effort was made to fast the fish immediately before the experiment. All experimental procedures were approved by the University of Alaska IACUC committee (570 217-9).

### Surgery and instrumentation

Anaesthesia was induced by immersing individual fish in seawater (15 l; at acclimation temperature) containing MS-222 (250 mg l^−1^). Once reflexes disappeared, fish were transferred to a surgical table and the gills were irrigated with seawater containing MS-222 (100 mg l^−1^). Surgery was conducted in an environmental room maintained at 4 ± 2°C. For 0°C-acclimated fish, the anaesthetic-containing seawater was chilled to 1°C with icepacks. The ventral aorta was dissected free at the base of the fourth gill arch to implant a Transonic (Transonic Systems Inc., USA) flow probe (2.5 or 4.0 mm diameter) immediately distal to the pericardium to measure *Q̇*. In five of the six 5°C-acclimated fish, and in all 0°C-acclimated fish, the left Ductus of Cuvier was non-occlusively cannulated with a PE-50 cannula, which was advanced into the sinus venosus (e.g. Altimiras and Axelsson, 2004; Sandblom *et al.*, 2008) allowing the measurement of *P*_cv_, an index of cardiac preload, and permitting the withdrawal of blood samples for measuring Hct. The flow probe lead and cannulas were then secured to the skin with 4–0 Prolene sutures.

### Experimental setup and data acquisition

**Figure 1:**
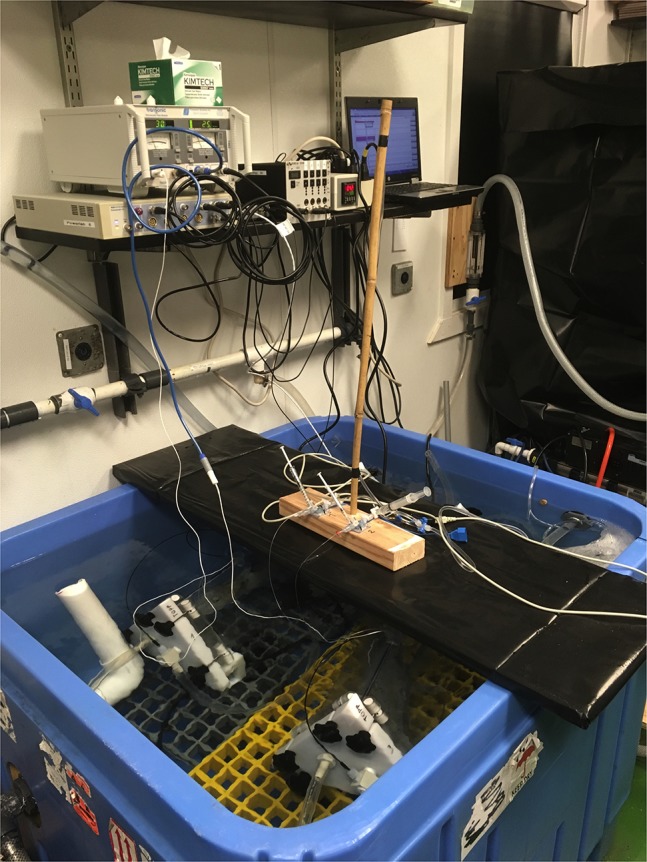
The experimental setup, consisting of two 12.4 l respirometers in a 700-l tank. During the experiment the tank was covered in black plastic to prevent disturbance

Immediately post-surgery, fish were transferred to one of the two 12.4-l, custom-made polyethylene terephthalate respirometers (PlastKapTek Sverige, Sweden) with a triangular cross-section, which were submersed in a 700-l tank held at each of the two respective acclimation temperatures (Joyce *et al.*, 2018; Fig. 1). Within the respirometer, the fish’s gills were flushed with seawater until spontaneous ventilation resumed.

Submersible pumps (Eheim, Germany) continually recirculated seawater in each respirometer, whilst a second pump introduced fresh seawater for 15 min during each 25 min period. For the remaining 10 min, oxygen partial pressure (PO_2_) was allowed to fall to permit the measurement of the rate of oxygen uptake (*Ṁ*_O2_). Water PO_2_ within the respirometer was continuously measured using a 3 mm robust FireSting optode connected to a fiberoptic O_2_ metre which was regularly calibrated *in situ* (FireSting, Aachern, Germany).

Flow probes were connected to a flow metre (Transonic; T402), and the cannulas connected to pressure transducers (Medizintechnik, Kirchseeon, Germany). Signals from the pressure transducers were pre-amplified using a Senselab 4ChAmp amplifier (Somedic sales, Hörby, Sweden). The pressure transducers were calibrated against a static water column prior to each experiment. The outputs from recording equipment were connected to a PowerLab system (ADinstruments, Castel Hill, Australia), which was connected to a computer running LabChart Pro (version 7; ADInstruments, Bella Vista, Australia)

### Experimental procedure

Cardio-respiratory measurements began after 48 h of post-surgical recovery (*cf*. Egginton, 1994). Resting parameters were first measured in 5°C-acclimated fish and 0°C-acclimated fish at their respective temperatures. Thereafter, the acute thermal challenge commenced. The fish acclimated at 5°C were initially cooled to 0°C over a period of 4 h. During heating, the fish were warmed at 2.6°C h^−1^ with a 3 kW titanium in-line heater (AquaLogic, San Diego, CA, USA) from 0°C to the temperature at which the heart failed (i.e. prolonged asystole; Joyce *et al.*, 2018), which we define as the critical thermal maximum (CT_max_). Heating was paused at 4°C increments for at least 20 minutes to measure oxygen consumption rate (*Ṁ*_O2_) at a steady temperature. Background respiration rate in blank respirometers was measured and was always negligible. Temperature in the holding tank was regularly measured off-line during each oxygen uptake measurement (i.e. every 20 min) and frequently (approximately every 5 min) at high temperature to allow precise logging of the temperature at which cardiac arrhythmia commenced (*T*_arr_) and the temperature of complete cardiac failure (CT_max_). In practise, complete cardiac failure occurred simultaneously with the loss of equilibrium (Joyce *et al.*, 2018; O’Brien *et al.*, 2018).

At the start of each experiment (at the fish’s acclimation temperature) as well as at the end (CT_max_), a 100-μl blood sample was withdrawn to measure Hct (in duplicate).

At the end of the experiments, fish were killed with a sharp blow to the head, the spinal cord was severed and the brain was destroyed by pithing. The fish were then de-instrumented and weighed. The ventricle was also weighed in all but one fish.

### Calculations and statistical analyses

Oxygen uptake (Ṁ_O2_) was calculated according to the equation:

$${\dot{M}}_{\mathrm{O2}}=((\alpha O_{2}\times V)\times \left(\Delta O_{2}/\Delta t\right))/\mathrm{Mb}$$

where αO_2_ is the oxygen content of sea water at a given temperature, is volume of the respirometer, ∆O_2_/∆*t* is the decline (change) in oxygen concentration (%) per unit time, and Mb is the fish body mass in kilogram.

Heart rate (*ƒ*_H_) was calculated automatically from the pulsatile *Q̇* trace.

*Q̇*, *ƒ*_H_ and *P*_cv_ were measured simultaneously with *Ṁ*_O2_ at 0, 4, 8, 12 and 16°C (the latter only applicable when fish remained viable). All of these cardiorespiratory parameters were also measured in 5°C-acclimated fish at 5°C before cooling to 0°C for comparison with 0°C-acclimated fish prior to heating. Flow probes were temperature compensated according to the manufacturer’s instructions.

Stroke volume (*V*_S_) was calculated according to the equation:

$$V_{s}=\dot{Q}/{ƒ}_{H}$$

Arterio-venous oxygen extraction was calculated according to the Fick equation

Oxygen extraction = *Ṁ*_O2_/*Q̇*

In fishes the Fick principle should be applied with caution because it does not account for cutaneous oxygen uptake, potentially leading to an overestimation of the arterio-venous oxygen difference (Farrell *et al.*, 2014). Nevertheless, we believe any possible error would be consistent between acclimation groups and the calculation therefore provides a valid and useful estimate.

Unpaired *t*-tests were used to investigate differences between experimental groups for *T*_arr_, CT_max_, body mass, ventricle mass, relative ventricular mass (RVM) and all five cardio-respiratory parameters (*Ṁ*_O2_, *Q̇*, *ƒ*_H_, *V*_S_ and *P*_cv_) in resting fish at acclimation temperatures prior to the acute thermal challenge. The ratio data (RVM) were arcsine transformed before analysis. To explore the effects of the variable acclimation times (6.0–9.5 weeks), linear regressions were used to investigate the relationship between duration of maintenance at 5°C and routine *Q̇*, routine *Ṁ*_O2_ and CT_max_. Two-way repeated measures analysis of variance (ANOVAs) were conducted to investigate changes in *Ṁ*_O2_, *Q̇*, *ƒ*_H_, *V*_S_ and *P*_cv_ at temperatures between 0 and 12°C. Sidak *post hoc* tests were used to compare differences between acclimation groups at each temperature and changes in each variable within each group at different temperatures. The repeated measures ANOVA could not be extended to 16°C due to sample size attrition resulting in incomplete matched data. However, to compare parameters at 16°C, separate one-way ANOVA’s and Tukey’s multiple comparisons tests were employed for the five fish in the 5°C-acclimated group that reached 16°C. A two-way ANOVA followed by Sidak post-hoc test was also used to investigate changes in Hct between experimental groups and before and after the acute temperature challenge.

Statistical significance was accepted at *P* < 0.05. All analyses were conducted in GraphPad Prism 7.0. All data are presented as individual values and/or mean ± standard error of the mean (SEM), except where stated otherwise.

## Results

Body mass, ventricle mass, and RVM are compared for 0°C- and 5°C-acclimated fish in Table 1. No significant differences existed for any of these parameters, although the numerically higher RVM in 5°C-acclimated fish approached significance (*t* = 2.2; df = 10; *P* = 0.053).

**Table 1:** Body mass, ventricular mass and relative ventricular mass in 0°C-acclimated or 5°C-acclimated *Notothenia coriiceps*

	Body mass (g)	Ventricular mass (g)	Relative ventricular mass (%)
0°C-acclimated	939.4 ± 38.4	0.92 ± 0.05	0.10 ± 0.003
5°C-acclimated	902.8 ± 86.2	1.06 ± 0.13	0.11 ± 0.004

*N* = 7 for 0°C-acclimated, and *N* = 6 for body mass and *N* = 5 for ventricular mass and relative ventricular mass in 5^o^C-acclimated fish. Data are mean ± SEM.

When measured at their acclimation temperature, *Ṁ*_O2_ and *Q̇* were significantly higher in 5°C-acclimated than 0°C-acclimated fish (Table 2; *t* = 2.3; df = 11; *P* < 0.05). The 2.7-fold higher *Q̇* (*t* = 6.3; df = 11; *P* < 0.05) was primarily attributable to a 2.2-fold increase in *V*_S_ (*t* = 8.3; df = 11; *P* < 0.05) and partially due to a much smaller (1.2-fold) increase in *ƒ*_H_ (*t*= 3.1; df = 11; *P* < 0.05). This increase in *V*_S_ was accompanied by a significantly increased *P*_cv_ in 5°C-acclimated fish (*t*= 4.6; df = 10; *P* < 0.05). Despite the 2.7-fold increase in *Q̇*, *Ṁ*_O2_ was increased by only 37% in 5°C-acclimated fish, meaning that the calculated arterio-venous oxygen extraction was significantly lower in the 5°C-acclimated than 0°C-acclimated fish (*t* = 4.7; df = 11; *P* < 0.05) (Table 1).

**Table 2:** Routine oxygen consumption (*Ṁ*_O2_), cardiac output (*Q̇*), heart rate (*ƒ*_H_), stroke volume (*V*_S_), central venous pressure (*P*_cv_) and arterio-venous oxygen extraction in 0^o^C- and 5^o^C-acclimated *Notothenia* prior to the acute temperature challenge.

	*Ṁ*_O2_ (mg O_2_ hour^−1^ kg^−1^)	*Q̇* (ml min^−1^ kg^−1^)	*ƒ*_H_ (beats min^−1^)	^*V*^_S_ (ml kg^−1^)	*P*_cv_ (kPa)	Oxygen extraction (mg O_2_ ml^−1^)
0^o^C-acclimated	42.3 ± 2.1	6.5 ± 0.5	25.2 ± 1.0	0.26 ± 0.02	0.08 ± 0.01	0.11 ± 0.01
5^o^C-acclimated	57.8 ± 7.0^*^	17.4 ± 1.8^*^	29.5 ± 0.8^*^	0.56 ± 0.03^*^	0.15 ± 0.01^*^	0.06 ± 0.01^*^

*N* = 7 for 0^o^C-acclimated, and *N* = 6 for 5^o^C-acclimated fish except for *P*_cv_ (*N* = 5). Values are mean ± SEM.

*Significant differences between groups (*t*-test; *P* < 0.05).

**Figure 2:**
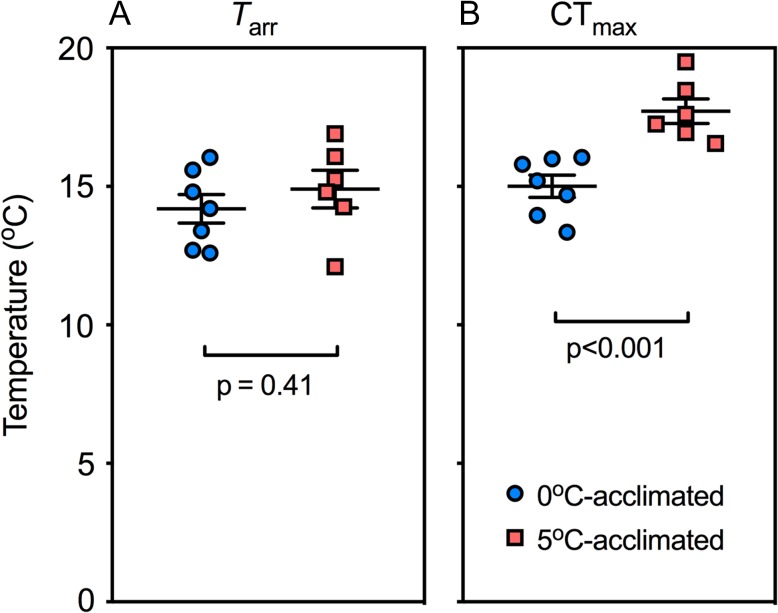
The effects of 0 and 5°C acclimation (6–9.5 weeks) on the temperature at which cardiac arrhythmia develops (*T*_arr_) and complete cardiac failure (CT_max_) occurs during an acute thermal challenge. Significant differences were evaluated with unpaired *t*-tests (*P* < 0.05). Data are mean ± SEM and individual data points

**Figure 3:**
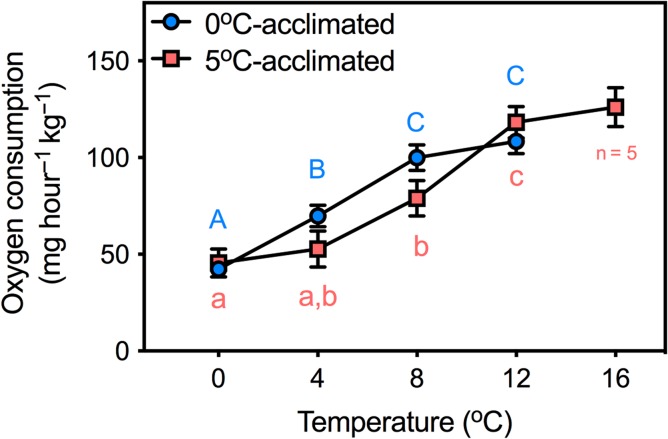
The effects of 0 and 5°C acclimation (6–9.5 weeks) on oxygen consumption during an acute thermal challenge. Significant differences were evaluated with two-way ANOVA. Dissimilar letters indicate significant differences within an acclimation group. There were no significant differences between acclimation groups at any temperature. *N* = 7 for 0°C-acclimated fish and *N* = 6 for 5°C-acclimated fish (except at 16°C where *N* = 5). Data are mean ± SEM

During acute warming, *T*_arr_ was not significantly different between the temperature acclimation groups (*t* = 0.85; df = 11; *P* = 0.41), but 5°C-acclimated fish sustained cardiac activity to a higher temperature, as revealed by the significantly higher CT_max_ (17.7 *vs*. 15.0°C; *t* = 4.6; df = 11; *P* < 0.05, Fig. 2). In the majority of 0°C-acclimated fish, cardiac performance collapsed at temperatures <16°C; thus, no cardio-respiratory variables are presented at 16°C in Fig. 3. However, in the 5°C-acclimated fish, five of the six animals maintained oxygen uptake and cardiac activity as high as 16°C.

During acute warming, *Ṁ*_O2_ at equivalent test temperatures was not significantly different between the two temperature acclimation groups (Fig. 3). However, the dynamics of how *Ṁ*_O2_ changed within each acclimation group during progressive warming revealed a divergence. Between 0 and 4°C, *Ṁ*_O2_ did not significantly change in 5°C-acclimated fish groups (*t* = 0.75; df = 33; *P* = 0.97) but increased significantly in 0°C-acclimated fish (*t* = 3.1; df = 33; *P* < 0.05) (Fig. 3). From 4 to 8°C, *Ṁ*_O2_ increased by a similar magnitude in both acclimation groups. However, *Ṁ*_O2_ peaked in 0°C-acclimated fish between 8 and 12°C (i.e. *Ṁ*_O2_ did not change from 8 to 12°C; *t* = 0.95; df = 33; *P* = 0.92), whereas it continued to increase in 5°C-acclimated fish (*t* = 4.1; df = 33; *P* < 0.05) before reaching a peak between 12 and 16°C. According to a separate one-way ANOVA conducted on the 5°C-acclimated fish that reached 16°C, *Ṁ*_O2_ did not change significantly from 12 to 16°C (*t* = 0.10; df = 4; *P* = 0.94).

**Figure 4:**
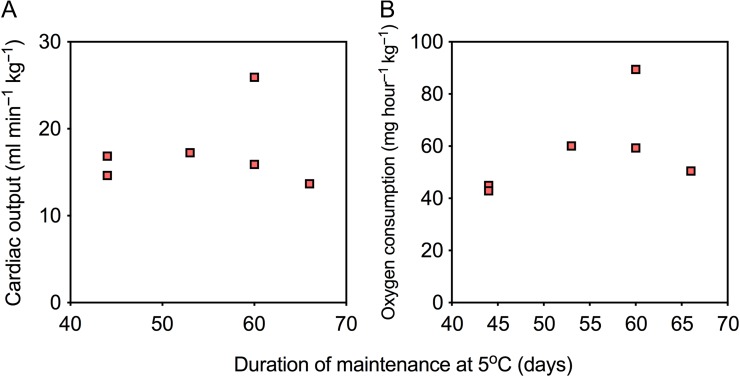
The relationships between acclimation duration and routine cardiac output (**A**) and oxygen consumption (**B**) at 5°C. As there were no significant effects of acclimation time on either variable, data for the 5°C acclimation group were pooled, irrespective of acclimation duration

**Figure 5:**
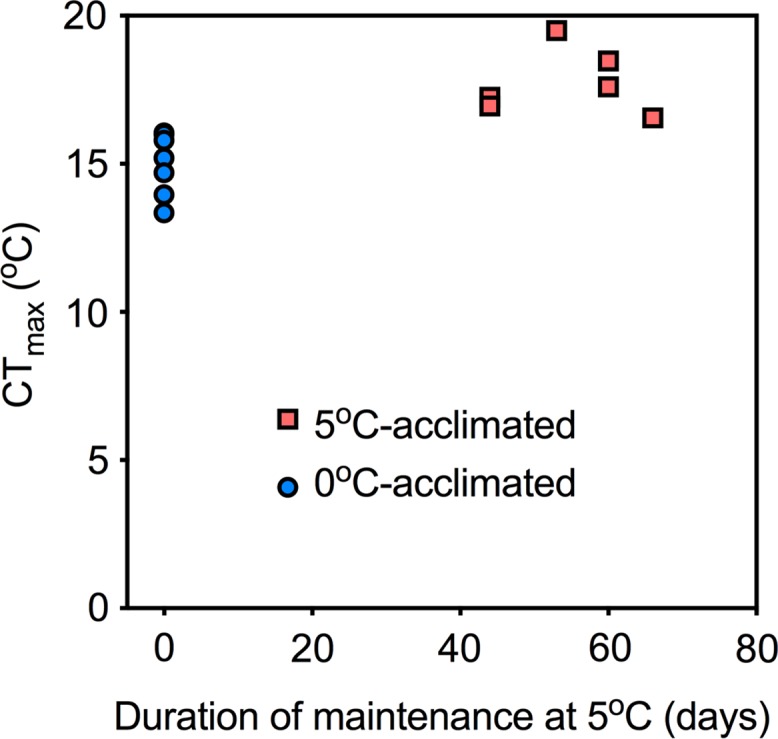
The relationship between acclimation duration and CT_max_ (defined as complete cardiac failure). As there was no significant effect of acclimation time on CT_max_, data for the 5°C acclimation group were pooled, irrespective of acclimation duration

There was no significant relationship between *Ṁ*_O2_ (*R*^2^ = 0.25, *P* = 0.31) or *Q̇* (*R*^2^ = 0.02, *P* = 0.77) and acclimation duration at 5°C (from 6.0 to 9.5 weeks; Fig. 4), and there was also no relationship between CT_max_ (the most easily discernible effect of acclimation) and acclimation time at 5°C (*R*^2^ < 0.001, *P* = 0.99) (Fig. 5). Therefore, all data for 5°C-acclimated fish were pooled, disregarding the variable acclimation periods.

**Figure 6:**
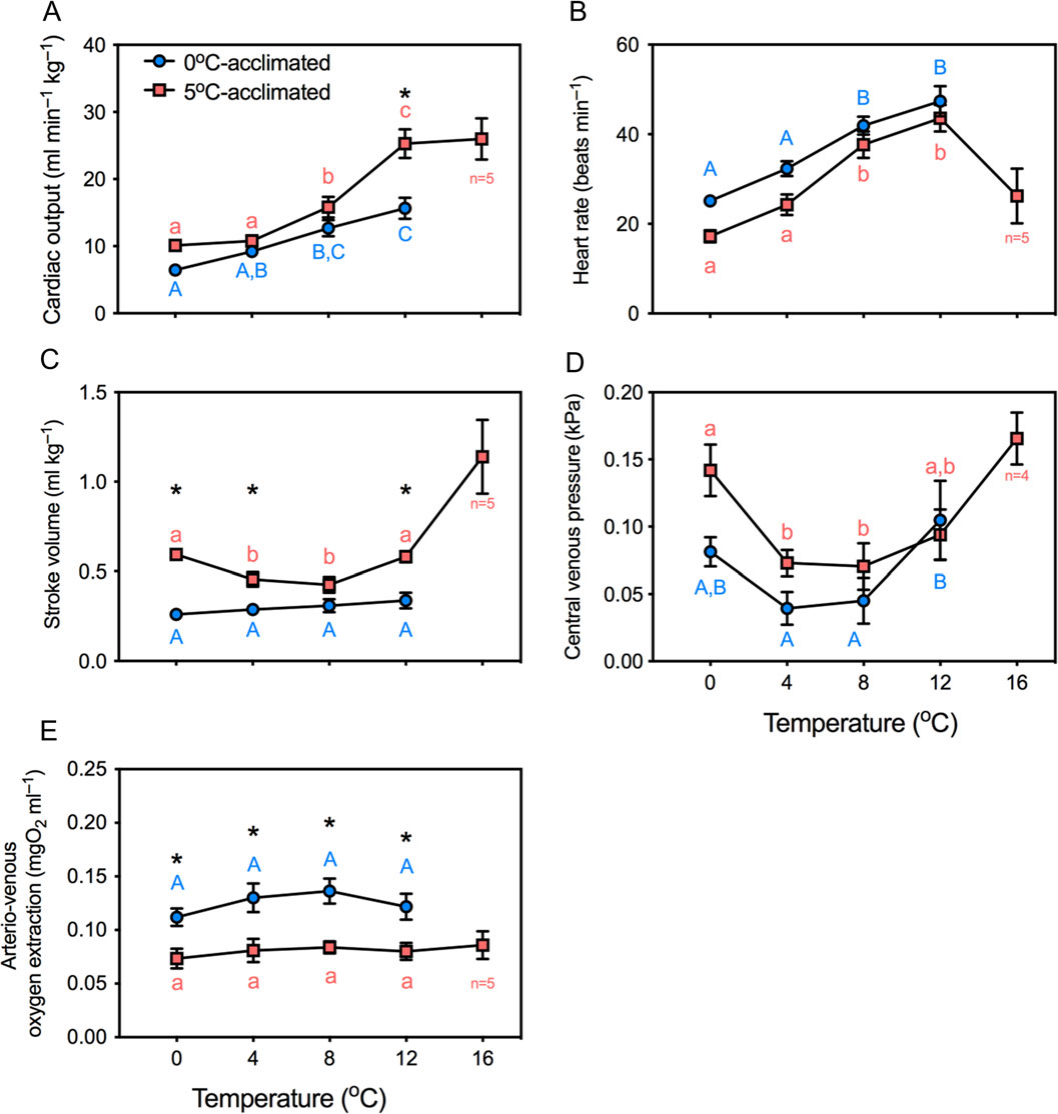
The effects of 0 and 5°C acclimation (6–9.5 weeks) on cardiac output (**A**), heart rate (**B**), stroke volume (**C**), central venous pressure (**D**) and arterio-venous oxygen extraction (**E**), during an acute thermal challenge. Significant differences were evaluated with two-way ANOVA. Dissimilar letters indicate significant differences within an acclimation group. Asterisks indicate significant differences between acclimation groups at a given temperature. *N* values same as for Fig. 3 except central venous pressure where *N* = 5 for 5°C-acclimated fish (*N* = 4 at 16°C). Data are mean ± SEM

During acute warming, *Q̇* measured at equivalent temperatures was not significantly different between temperature acclimation groups, with the exception of a higher *Q̇* in 5°C-acclimated animals at 12°C (Fig. 6A; *t* = 5.5; df = 44, *P* < 0.05). This was a result of a greater *V*_S_ (*t* = 5.2; df = 44; *P* < 0.05), while *ƒ*_H_ was not statistically different (*t* = 1.1; df = 44; *P* = 0.7) (Fig. 6B and C). *P*_cv_ measured at equivalent temperatures was not significantly different between thermal acclimation groups. *P*_cv_ decreased significantly as temperature increased from 0 to 8°C but then increased above 8°C in both acclimation groups (*P* < 0.05 in all cases, Fig. 6D). In the 5°C-acclimated fish that reached 16°C, *Q̇* was maintained from 12°C (*t*= 0.28; df = 4; *P* = 0.99).

**Figure 7:**
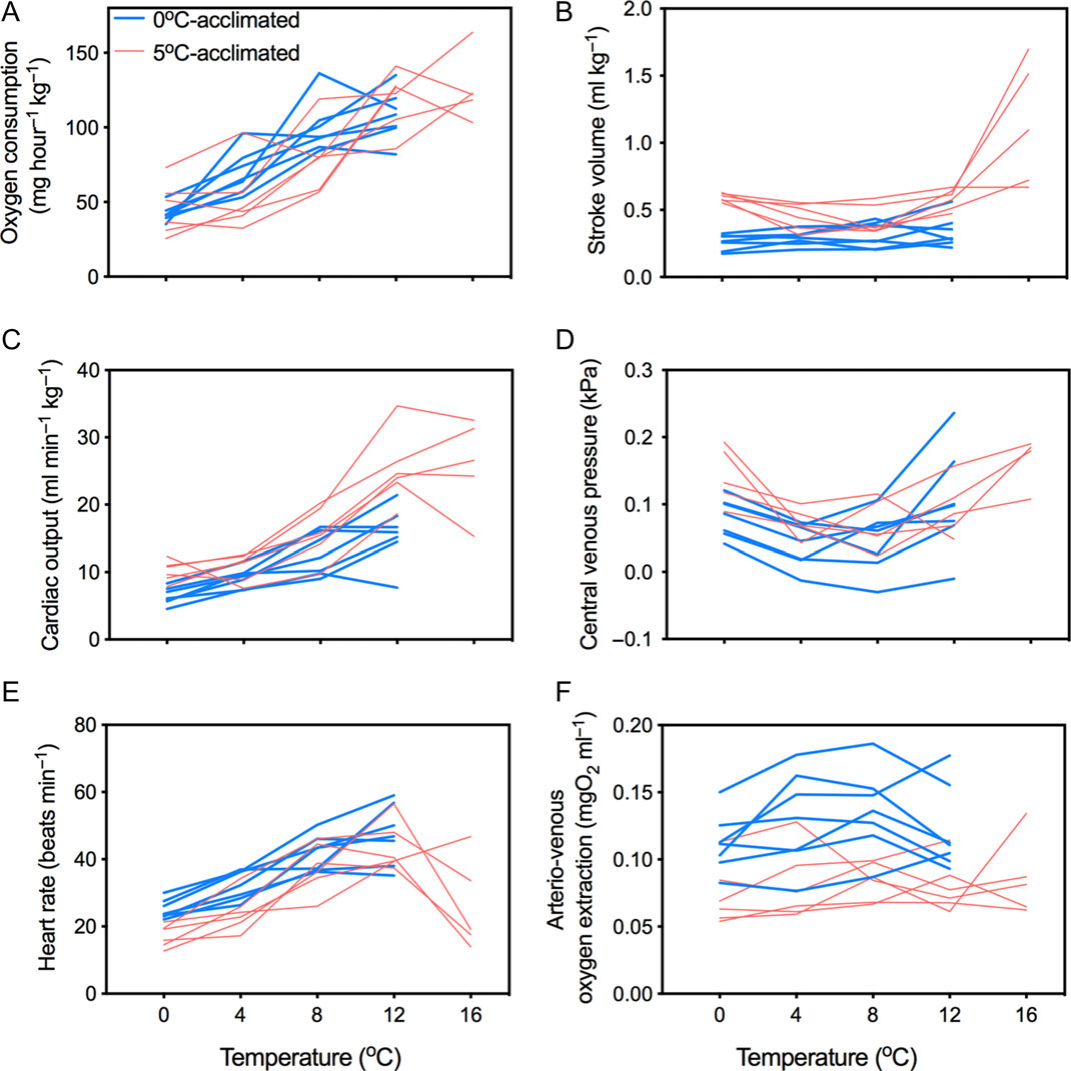
Individual data curves demonstrating the effects of 0°C (blue lines) and 5°C acclimation (6–9.5 weeks, red lines) on oxygen consumption (**A**), stroke volume (**B**), cardiac output (**C**), central venous pressure (**D**), heart rate (**E**) and arterio-venous oxygen extraction (**F**) during an acute thermal challenge

In order to illustrate inter-individual variation, the cardio-respiratory variables during acute warming are plotted from each individual in Fig. 7. In four of the five 5°C-acclimated fish (Fig. 7), the transition from 12 to 16°C was associated with bradyarrhythmia (as it occurred after *T*_arr_) and an increase in *V*_S_, although neither *f*_H_ (*t*= 3.8; df = 4; *P* = 0.22) nor *V*_S_ (*t* = 3.5; df = 4; *P* = 0.27) changed significantly due to the inter-individual variability. Arterio-venous oxygen extraction remained significantly higher in 0°C-acclimated fish than in 5°C-acclimated fish at each temperature, although it did not change significantly during warming in either group (Figs 6E and 7F).

**Figure 8:**
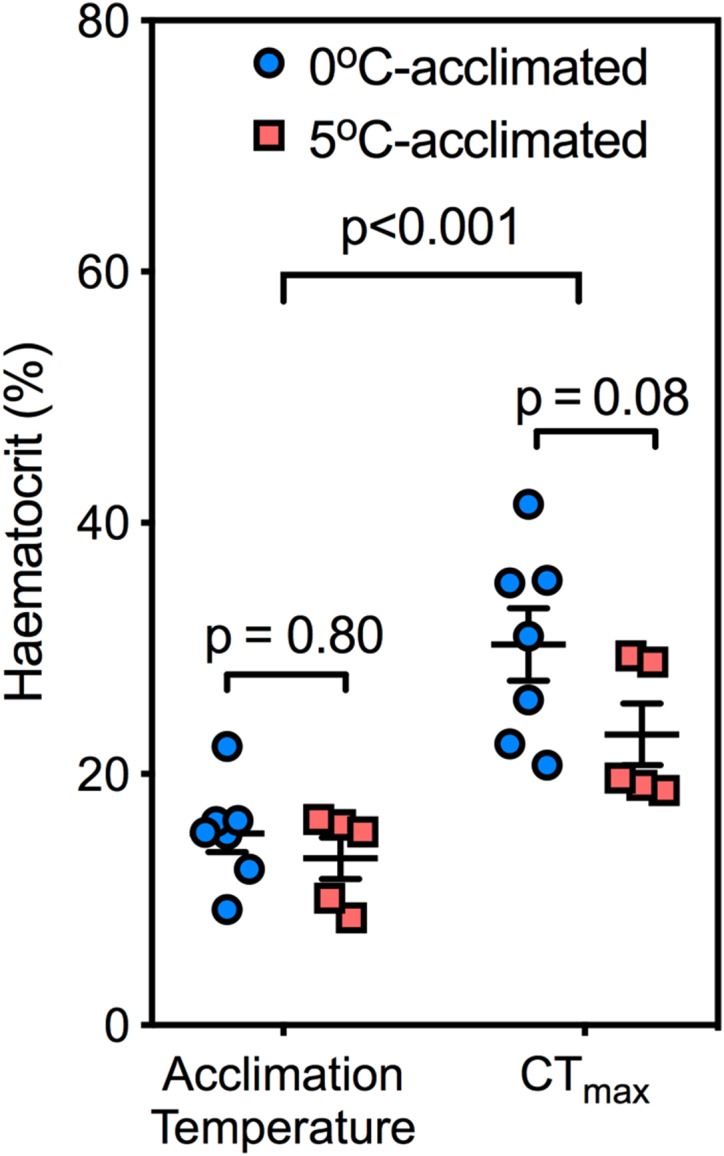
The effects of 0 and 5°C acclimation (6–9.5 weeks) on haematocrit at acclimation temperature and upon reaching CT_max_. Significant differences were investigated with a two-way ANOVA. Data are mean ± SEM and individual data points

There were no significant differences in Hct between 0°C- and 5°C-acclimated fish prior to the temperature challenge (Fig. 8; *t* = 0.8, df = 20, *P* = 0.80). Nevertheless, this parameter doubled in both acclimation groups by the time fish had reached their CT_MAX_ (0°C-acclimated fish: *t* = 7.1, df = 10, *P* < 0.05; 5°C-acclimated fish: *t* = 3.9, df = 10, *P* < 0.05).

## Discussion

The ability of Antarctic fishes to tolerate rising temperatures may be key to their fate in the Southern Ocean as it continues to warm. Given the rapid nature of the present warming, phenotypic plasticity (i.e. acclimation potential) will likely be of crucial importance. Our results suggest that for the red-blooded, benthic notothenioid *N. coriiceps* some aspects of its cardio-respiratory performance (e.g. cardiac CT_max_) are capable of changing in response to acclimation to 5°C. However, even after 9.5 weeks of exposure to 5°C, *Ṁ*_O2_ showed no sign of down-regulation. Thus, survival at elevated temperature comes with the cost of chronically elevated oxygen requirements (approximately a third higher than at 0°C), which would require increased food intake and likely increased costs associated with foraging behaviour. It remains plausible and unknown whether or not a longer acclimation period would result in a compensation of *Ṁ*_O2_. Unfortunately, such studies may prove to be a logistical challenge given the remoteness of Antarctic field stations and the confines of limited field seasons.

The temperature at the onset of cardiac arrhythmia (*T*_arr_) in *N. coriiceps* was unaffected by acclimation to 5°C for 6.0–9.5 weeks, which is consistent with data from a high latitude Antarctic notothenioid, *T. bernacchii* (Jayasundara *et al.*, 2013; 2 weeks of warm acclimation), but in contrast to reports of temperate fish species (Jayasundara and Somero, 2013; Ferreira *et al.*, 2014), which demonstrate an increase in *T*_arr_ following 3–4-week acclimation regimes. Together, our data suggest that in Antarctic notothenioid fishes, the mechanisms responsible for action potential propagation and electrical conduction either are not plastic or require >9.5 weeks of acclimation. However, despite arrhythmias, the hearts of 5°C-acclimated fishes retained residual (arrhythmic) function to a higher temperature (i.e. CT_max_ was higher) than the animals held at 0°C. These results indicate that the thermally-sensitive mechanisms responsible for ultimately sustaining cardiac pumping are not necessarily the same as those involved in electrical conduction. In brown trout (*Salmo trutta fario*) it has been demonstrated that voltage-gated Na^+^ channel function, which largely determines action potential propagation (Vornanen, 2017), is the ‘weak-link’ in thermally-stressed fish heart (Vornanen *et al.*, 2014), which is consistent with our finding that conduction began to fail before pumping capability. Furthermore, in electrocardiogram-instrumented *N. coriiceps*, we clearly observed conduction failure (atrio-ventricular block) before reaching CT_max_ (Joyce *et al.*, 2018). The ability to sustain *Q̇* at higher temperatures supports our hypothesis that whole animal thermal tolerance (i.e. CT_max_ determined by loss of righting reflex; Bilyk and DeVries, 2011) extended by warm acclimation is associated with improved thermal tolerance of cardiac function.

An important and surprising discovery of our study was that 5°C-acclimated *N. coriiceps* had a greater *Q̇* than 0°C-acclimated *N. coriiceps* at their acclimation temperatures, predominantly due to a doubling of *V*_S_ with only a small increase in *f*_H_. This is in direct contrast with the effects of acute warming in fish, in which it has been consistently demonstrated that augmented *Q̇* is achieved by an increase in *ƒ*_H_ (reviewed by Farrell, 2016, and evident during our acute warming experiment). Thus, our hypothesis that the elevated oxygen demands would also be primarily supported by increased *ƒ*_H_ is not supported by our findings.

It is unlikely that the 2-fold increase in *V*_S_ was due to the modest (10%) increase in RVM, but it is more likely associated with the increased cardiac preload (*P*_cv_). The association between the increases in *V*_S_ and *P*_cv_ in 5°C-acclimated fish is consistent with a classic Frank–Starling mechanism, which describes the greater force generated by a more distended myocardium (Patterson and Starling, 1914; Shiels and White, 2008). The precise mechanism responsible for the elevated *P*_cv_ remains to be determined, but it is well-established that blood volume is a key determinant of cardiac filling in fishes (Sandblom and Axelsson, 2006; 2007; Sandblom and Gräns, 2018). In the brook trout (*Salvelinus fontinalis*), blood volume increased over 25% following acclimation from 2 to 5°C (Houston, DeWilde MA, 1969). Although we are not aware of equivalent work in Antarctic fishes, Petzel (2005) reported a profound (460%) increase in drinking rate in 5°C-acclimated *T. bernacchii*. This is primarily to compensate for the decreased serum osmolarity, which promotes water loss to hyperosmotic seawater (Petzel, 2005). Increasing *V*_S_ through *P*_cv_ (and presumably blood volume) may be compatible with increased osmotic water loss. For example, it has recently been demonstrated that during the freshwater to seawater transition in rainbow trout, *V*_S_ increases as a result of increased *P*_cv_ (Brijs *et al.*, 2017), which is likewise coincident with increased drinking in the face of increased osmotic water loss. Thus, we predict that an increase in blood volume may be a fundamental change following thermal acclimation in *N. coriiceps*. The regulation of blood volume and cardiac filling in Antarctic fishes, particularly following different thermal acclimations, remains an interesting avenue for future investigation.

At all equivalent test temperatures during acute warming, *Ṁ*_O2_ was the same for 5°C-acclimated and 0°C-acclimated fish, a result that is consistent with reports in the same species following acute (1 day) and chronic (6 weeks) acclimation to 5°C (Egginton and Campbell, 2016). However, we discovered some aspects of oxygen uptake that were different between 5°C-acclimated and 0°C-acclimated fish. For example, in 5°C-acclimated fish, ^*Ṁ*^_O2_ was thermally independent between 0 and 4°C during acute warming, in contrast with the thermal sensitivity of *Ṁ*_O2_ in fish 0°C-acclimated fish, which is akin to the response observed in Atlantic halibut (*Hippoglossus hippoglossus*) (Gräns *et al.*, 2014). Another difference between acclimation groups is that both *Ṁ*_O2_ and *Q̇* peaked between 8 and 12°C in fish 0°C-acclimated, yet not in 5°C-acclimated fish. Together, these data demonstrate divergent responses to warming between acclimation groups at sub-lethal temperatures (i.e. <12°C) (e.g. Pörtner and Knust, 2007; Pörtner and Farrell, 2008).

Our measurements of resting Hct (~15%) are consistent with previous reports in *N. coriiceps* that were likewise instrumented with indwelling cannulae (Egginton, 1994; 1997). At their respective acclimation temperatures, we did not observe a difference in Hct between 0°C-acclimated and 5°C-acclimated animals. Optimal Hct represents a compromise between the advantages gained from increased oxygen carrying capacity of blood with the burden of increasing blood viscosity, which elevates peripheral resistance and requires greater cardiac work to pump blood (Gallaugher *et al.*, 1995). Because increasing temperature increases *Ṁ*_O2_ (Fig. 3) while at the same time decreases the viscosity of blood (Egginton, 1996) and water oxygen concentration falls, it could be expected that a higher Hct may be favoured at higher temperature. Indeed, Tetens *et al.* (1984) observed that warm acclimation of *P. borchgrevinki* significantly increased Hct from 15 to 22%. However, our finding that Hct did not change following warm acclimation in *N. coriiceps* is in agreement with another study on *P. borchgrevinki* (Lowe and Davison, 2005) and *T. bernacchii* (Hudson *et al.*, 2008). Lowe and Davison (2005) ascribed the discrepancy between their findings and those of Tetens *et al.* (1984) as an effect of prolonged fasting, but this does not hold true for the fish in our study that continued to feed throughout the acclimation period. Our data, rather, suggest that the increase in *Q̇* (Table 1) is sufficient to maintain the chronically increased routine *Ṁ*_O2_ without an increase in Hct.

With acute warming, Hct doubled in both 5°C-acclimated and 0°C-acclimated fish, consistent with a much smaller effect observed previously in *N. coriiceps* (Hct increased from ~35% at 0°C to 41% in animals at their CT_MAX_; Beers and Sidell, 2011). However, resting Hct of *N. coriiceps* in the previous study (Beers and Sidell, 2011) was markedly higher than our Hct value, probably due to the stress associated with capture and anaesthesia (Wells *et al.*, 1984) prior to acutely sampling blood from the caudal vein, likely masking some of the effect of the temperature response. This suggests that *N. coriiceps* is indeed capable of significant, and previously vastly underestimated, changes in Hct during acute warming, as previously reported in other Antarctic notothenioids (*P. borchgrevinki*: and *T. bernacchii*: Franklin *et al.*, 1991; Davison *et al.*, 1994).

In a eurythermal temperate species, the European perch (*Perca fluviatilis*), Sandblom *et al.* (2016) observed that *Ṁ*_O2_ and *Q̇* increased approximately in parallel following chronic warm acclimation (22–23 *vs.* 17–18°C), suggesting that arterio-venous oxygen extraction was largely unchanged. In contrast, we observed that 5°C-acclimated fish exhibited 2-fold lower arterio-venous oxygen extraction values than 0°C-acclimated fish, both at acclimation temperature and during acute warming. Although the mechanistic basis for this was beyond the scope of our study, it is possible that the increased *Q̇* in 5°C-acclimated fish exceeded the peripheral oxygen utilization capacity and *Ṁ*_O2_ was limited by mitochondrial oxidative capacity (see Wagner, 2011). Limited peripheral oxygen utilization may represent a general constraint for the warm acclimation of stenothermal species, a hypothesis that deserves future exploration. We observed that arterio-venous oxygen extraction did not change in either acclimation group during acute warming, which is consistent with other teleosts such as Sockeye salmon (*Oncorhynchus nerka*) (Steinhausen *et al.*, 2008) and European eel (*Anguilla anguilla*) (Claësson *et al.*, 2016) in the resting state.

Physiological studies to assess the chronic and acute responses to warming are key in predicting the effects of a changing climate on animal distribution and performance (Seebacher *et al.*, 2014, Deutsch *et al.*, 2015). The finding that some, but not all, components of cardio-respiratory performance are changed (i.e. CT_max_ increased whereas *T*_arr_ did not change) following warm acclimation is in-line with the previous mixed results that have typified work on acclimating Antarctic notothenioid fish to elevated temperature (e.g. Franklin *et al.*, 2007; Jayasundara *et al.*, 2013; Egginton and Campbell, 2016). It is probable that previously conflicting results may be accounted for by differences in species and acclimation regimes. A more comprehensive insight into the capacity for Antarctic notothenioids to adapt to a changing climate may only be achieved with interspecific investigations that adopt standardized and sufficiently long-term acclimation protocols. One of the most pertinent outcomes of our study is that, even without warm acclimation, *N. coriiceps* is capable of robust cardiovascular performance at temperatures well in excess of current environmental temperatures, and likely in the near future temperatures (i.e. the 2°C rise above ambient predicted in the next 100 years; IPCC, 2014). This is consistent with other studies that have revealed a surprisingly broad thermal tolerance for some traits, for example, burst swimming, in Antarctic fishes (Wilson *et al.*, 2001). However, given the need to maintain elevated *Ṁ*_O2_, ecological factors (e.g. changes in species assemblages and food availability; Ashton *et al.*, 2017), or food assimilation and reproductive capacity (Sandersfeld *et al.*, 2015), may prove to be more critical considerations than intrinsic limitations of cardio-respiratory oxygen transport.
